# Emerging infectious diseases

**DOI:** 10.1016/j.mpmed.2013.10.014

**Published:** 2014-01

**Authors:** H. Rogier van Doorn

**Affiliations:** **H Rogier van Doorn MSc (Med) MSc (Med Biol) MD PhD** is a Clinical Microbiologist from Amsterdam, The Netherlands, who works as a Researcher and Group Head of Emerging Viral Infections at the Oxford University Clinical Research Unit in Ho Chi Minh City, Viet Nam. Conflicts of interest: none declared

**Keywords:** drivers of emergence, emerging infections, hotspots for emergence, species jump, zoonosis

## Abstract

The spectrum of human pathogens and the infectious diseases they cause is continuously changing through evolution and changes in the way human populations interact with their environment and each other. New human pathogens most often emerge from an animal reservoir, emphasizing the central role that non-human reservoirs play in human infectious diseases. Pathogens may also re-emerge with new characteristics, such as multidrug-resistance, or in different places, such as West Nile virus in the USA in 1999, to cause new epidemics. Most human pathogens have a history of evolution in which they first emerge and cause epidemics, become unstably adapted, re-emerge periodically, and eventually become endemic with the potential for future outbreaks.

**What's new?**

• Infectious disease are continuously emerging
• Most known human pathogens are zoonoses
• Most that are not zoonoses have zoonotic origins
• Globalization and human invasiveness creates more opportunities for emergence
• Global surveillance and research consortia and novel technologies will allow for more frequent and more rapid detection of novel pathogens

## Introduction

In the 1970s, with antibiotics and vaccines at hand and the eradication of smallpox within reach, there was a general optimism that infectious diseases would soon be a thing of the past. ‘If […] we retain a basic optimism and assume no major catastrophes occur […] the most likely forecast about the future of infectious disease is that it will be very dull’.^1^ The pandemic of HIV crushed this optimism and infectious diseases were put back on the global health agenda of which the 1992 publication ‘Emerging Infections: Microbial Threats to Health in the United States’^2^ is a landmark. Since then, the ongoing antimicrobial resistance development among many different pathogens, the continuous emergence of (mostly) viruses with potential for human-to-human or pandemic spread, the intentional release of pathogens as terrorist weapons and the heated debates about experiments to make avian influenza viruses transmissible in ferrets are continuously reminding us that infectious diseases are far from dull.

## Definitions

‘Emerging infectious diseases’ are defined as ‘those whose incidence in humans has increased within the past two decades or threatens to increase in the near future. Emergence may be due to the spread of a new agent, to the recognition of an infection that has been present in the population but has gone undetected, or to the realization that an established disease has an infectious origin. Emergence may also be used to describe the reappearance (or re-emergence) of a known infection after a decline in incidence’.^2^

## Zoonotic emergence

**Pathogen:** there are 1400 known human pathogens, the majority (60%) of which are transmitted to humans zoonotically and depend on an animal reservoir for their survival. An additional smaller proportion (5–10%) is environmentally transmitted, and the remainder consists of pathogens that can be maintained by an exclusively human-to-human transmission cycle. Among emerging infections, the proportion of zoonotic infections is even higher (73%), indicating that the human–animal interface presents a risk for emergence.^3^ In addition, almost all (now) established strictly human pathogens have zoonotic origins:^3^, ^4^ these pathogens have moved from animals into humans and fully adapted to them during many millennia of human and pathogen evolution.

**Human:** because most human pathogens rely on an animal or environmental reservoir, the interactions between human populations and their surrounding ecosystem determine the local pathogen spectrum, and the interpopulation interactions determine the spread of these pathogens. Historically, there have been several profound and distinct transitions in human environmental and interpopulation interactions that have radically changed the spectrum and causes of infectious disease in human populations (Table 1). Today, we are living through the fourth great historical transition. The invasiveness of human activity into all geographic areas of the world, the globalization of economic activities and culture, the speed and accessibility of distant contact, the spread and intensification of urbanization, and our increasing reliance on either intricate or massive technology, are reshaping the relations between humans and microbes.^5^

**Table 1:** Transitions in human environmental and interpopulation interactions through time

Transition, time	Major change
Prehistoric transition, millions of years ago	From tree-dwelling to savannah, hunter-gatherer
Historic transitions	
• first (local), 5000–10,000 years ago	Settlements, crop and livestock domestication
• second (continental), 1000–3000 years ago	Intracontinental military and commercial contacts
• third (intercontinental), from AD 1500	European exploration and imperialism
• fourth (global), today	Globalization, urbanization, climate change

**The species jump:** the species jump that initiates a first human infection by a new agent is often brought about by a novel or unusual physical contact between potential pathogen and human. Such contacts usually occur because of cultural, social, behavioural or technological change on the part of humans that affects the human–animal interface. The potential for subsequent spread of this ‘new’ infectious disease will depend on many different factors, including environmental or social factors. These changes and factors are the drivers of emergence and are listed in Table 2.^2^

**Table 2:** Biological, social and environmental drivers of emergence of infectious disease

• Microbial adaptation and change • Susceptibility to infection • Climate, weather and the environment • Economic development and land use • Human demographics and behaviour • Technology and industry • International travel and commerce • Breakdown in public health • Poverty and social inequality • War and conflict • Urban decay • Lack of political will • Intentional biological attacks

**Figure 1:**
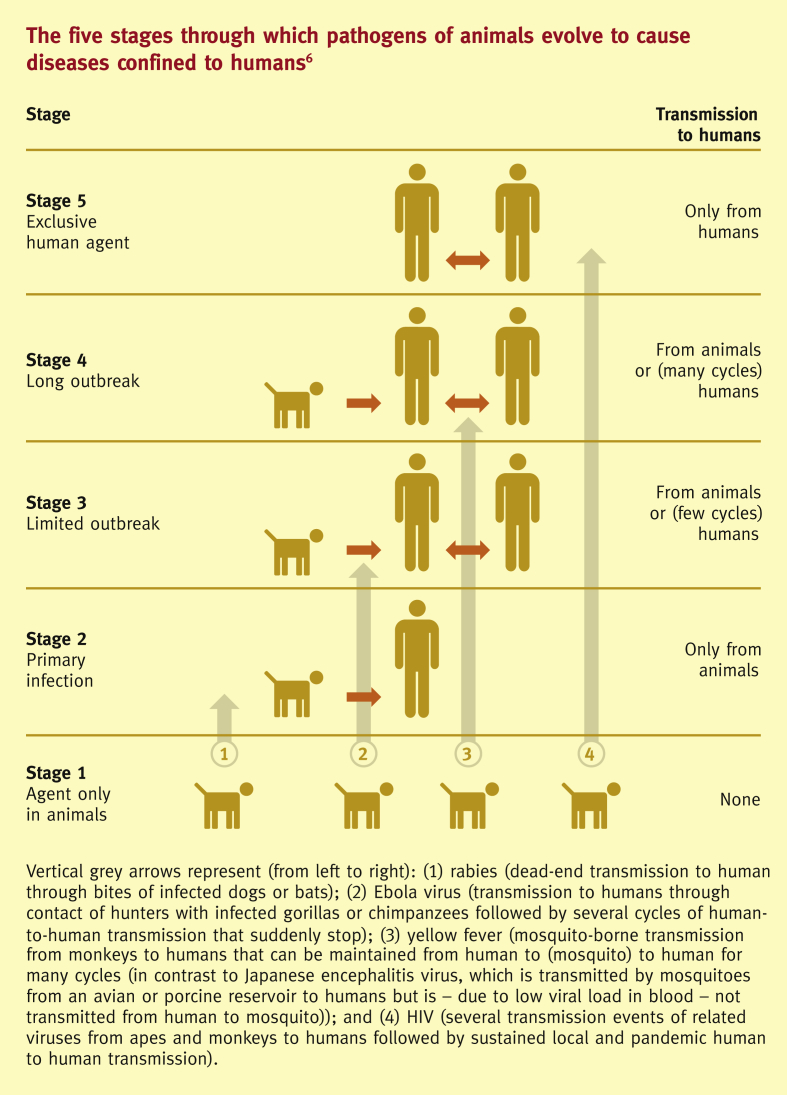
Adapted from Wolfe ND et al. Origins of major human infectious diseases. Nature 2007; 447 (7142).

**Figure 2:**
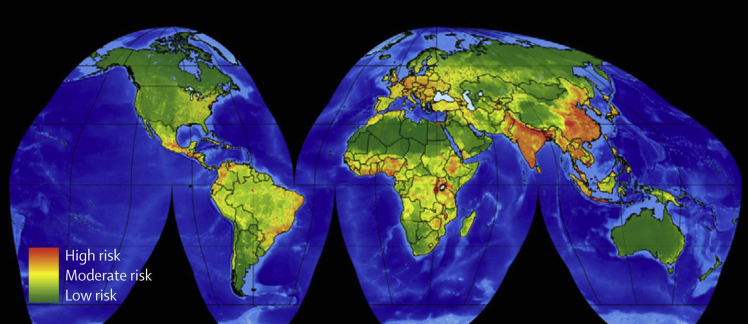
Global hotspots for emerging diseases originating in wildlife.

Biologically, the species jump is often more a transition process involving several stages rather than a single event. These stages are displayed in Figure 1.^6^ The pathogen has to overcome various biological barriers (interspecies, intrahuman and interhuman) to move from one stage to the next, to be able finally to cause sustained human-to-human transmission.^7^ Based on data from 1940 onwards, the hotspots for emergence of infectious diseases were mapped for zoonotic infections from wildlife and domestic animals, and for drug-resistant and vector-borne organisms. Figure 2 shows that these hotspots are primarily located in South and South East Asia, South and Central America and Subsaharan Africa.^8^, ^9^

Reprinted by permission from Macmillan Publishers Ltd.

From Morse SS, Mazet JA, Woolhouse M, Parrish CR, Carroll D, Karesh WB et al. Prediction and prevention of the next pandemic zoonosis. *Lancet*. 2012 Dec 1;**380**:1956–65. With permission from Elsevier.

Various international consortia and large research programmes have been established in an attempt to predict and prevent, or prepare for and mitigate, these novel emergence events, summarized in a recent issue of *The Lancet*.^8^ Technical advances enable us to detect and characterize these agents much more rapidly than ever before (e.g. availability of whole genome sequences of influenza virus A/H7N9 influenza or *Escherichia coli* O104:H4 within days).^10^, ^11^

## Non-zoonotic emergence

The emergence of novel zoonotic pathogens is appealing to the imagination and draws plenty of popular and scientific media attention, but does not necessarily represent the largest threat from infectious diseases. There is a rapid and increasing spread of antimicrobial drug resistance among bacteria and other pathogens, and the development of novel antimicrobial agents has almost come to a stop because drug companies do not consider them profitable: a combination that may set us back to the pre-antibiotic era. Drug resistance is a threat not only to the successful treatment of HIV, malaria and tuberculosis, but also, increasingly, of hospital- and community-acquired infections from ‘normal’ Gram-positive and Gram-negative bacteria. Failure of vaccination programmes because of bad press or religious conviction in developed countries can cause re-emergence of highly infectious viruses, such as those that cause measles or rubella, within years, as has happened in the UK and the Netherlands. Global food production and distribution processes may give rise to widely disseminated foodborne infections that are hard to tackle, as with *E. coli* O104:H4 in and out of Germany, recently. Finally, in South East Asia, while H5N1 and H7N9 influenza viruses attract most international attention, hand, foot and mouth disease, caused by the exclusively human pathogen, enterovirus 71, is now associated annually with hundreds of thousands of hospitalizations of children under 5, with a mortality of around 0.1%,^12^ showing that humans can also be a source of emerging infections (Table 3).

**Table 3:** Selection of important emerging infectious diseases from the last decade

2013	Influenza virus A/H7N9
2012	Middle East respiratory syndrome (MERS) – coronavirus
2011	*Escherichia coli* 0104:H4
2010	Huaiyangshan virus, associated with severe fever and thrombocytopenia syndrome (SFTS)
2009	Influenza virus A/H1N1pdm09
2008	*Plasmodium knowlesi*
	Lujo virus
2005	Human retroviruses HTLV3 and HTLV4
2004	Re-emergence of influenza virus A/H5N1
2003	SARS coronavirus

## Conclusion

For daily medical practice it is important for doctors, and especially infectious disease physicians, to be aware of events of emergence and countries where processes of emergence and species-jumping are occurring (e.g. by subscribing to ProMED, WHO influenza update or others). It is crucial that for each patient the history should include a travel history, which involves more than asking merely for the name of the country that a patient has visited. In the end, despite sophisticated surveillance programmes, it is usually an astute clinician who, after having seen or heard one or two extraordinary patient histories, makes the connection and sees the first signs of an event of emergence.
